# The Role of Wearables in Heart Failure

**DOI:** 10.1007/s11897-020-00467-x

**Published:** 2020-06-03

**Authors:** Arvind Singhal, Martin R. Cowie

**Affiliations:** 1 Royal Brompton Hospital, London, UK; 2 National Heart and Lung Institute, Imperial College London, Dovehouse Street, London, SW3 6LY UK

**Keywords:** Wearables, Digital health, M-health, Apps; heart failure

## Abstract

**Purpose of Review:**

This review discusses how wearable devices—sensors externally applied to the body to measure a physiological signal—can be used in heart failure (HF) care.

**Recent Findings:**

Most wearables are marketed to consumers and can measure movement, heart rate, and blood pressure; detect and monitor arrhythmia; and support exercise training and rehabilitation. Wearable devices targeted at healthcare professionals include ECG patch recorders and vests, patches, and textiles with in-built sensors for improved prognostication and the early detection of acute decompensation. Integrating data from wearables into clinical decision-making has been slow due to clinical inertia and concerns regarding data security and validity, lack of evidence of meaningful impact, interoperability, regulatory and reimbursement issues, and legal liability.

**Summary:**

Although few studies have assessed how best to integrate wearable technologies into clinical practice, their use is rapidly expanding and may support improved decision-making by patients and healthcare professionals along the whole patient pathway.

## Introduction

Heart failure (HF) affects approximately 1–2% of the population in high-income countries [1, 2], and its prevalence is increasing [3]. Over 30% of patients seen in the clinic setting, and over 40% of those recently admitted with decompensation, will have a further hospitalisation within 12 months [4]. This high readmission rate, despite current approaches to disease management and monitoring, suggests that the traditional model of outpatient HF care, in which decisions are made about management based on data taken from a “snapshot” during short clinic visits, often many months apart, is in need of modernisation.

Advances in technology provide potentially new solutions to HF care: for example, titrating diuretics and other drug therapy based on pulmonary artery pressure data from an implantable monitor that can be interrogated remotely (CardioMEMS™ device) significantly reduces HF hospitalisation in patients with at least moderately severe symptoms [5•]. Such devices, of course, require a minimally invasive implant and are currently expensive, which limits their wider uptake. They also require changes to the workflow for clinical teams remotely monitoring the data and taking action in response to such data. Meanwhile, the direct-to-consumer health technology industry is thriving (now worth billions of dollars annually), and nearly 20% of Americans report using fitness monitors to track their health statistics [6]. These wearable devices, although chiefly marketed for “health and fitness” purposes, are becoming increasingly sophisticated and accurate and can be considered biological sensors. The remote detection and monitoring of physiological signals should, at least in theory, permit a more personalised and empowered experience for the person living with HF, with more frequent and appropriate adjustment of therapy and care, and the earlier detection of signals that might presage decompensation.

**Fig. 1:**
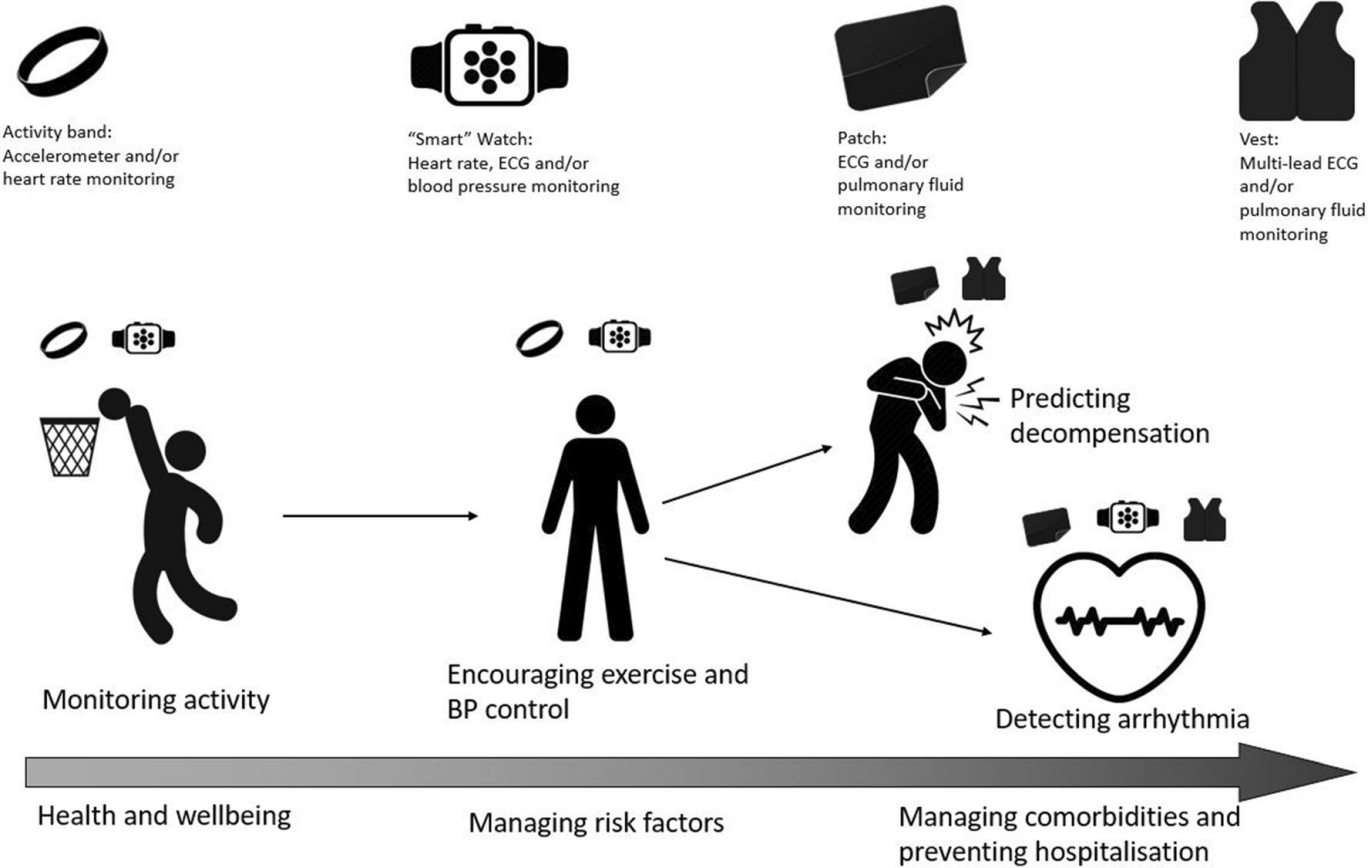
Examples of wearable devices used in health and cardiovascular disease

This review explores the current evidence base for wearable devices in HF and the challenges and opportunities that these bring for both the “consumer” and the healthcare professional. We define a wearable device as a non-invasive sensor that is worn, i.e. a device that is externally applied to the body, measures a signal and collects these data which can then be transmitted and/or stored for further analysis and decision-making. Such devices are often described as “smart” accessories or clothing; Fig. 1 shows examples of wearable devices that may be used at different points in the HF “journey” from prevention through to detection of decompensation.

## How Do Wearables Integrate into the Healthcare System?

Many devices that can monitor physical activity, heart rate, blood pressure and even the electrocardiogram (ECG) are currently marketed to consumers, although such data may potentially also be used by clinicians to guide decision-making and lifestyle and/or drug management. Wearable medical devices being developed for healthcare professionals primarily focus on predicting acute decompensated heart failure or confirmation of intermittent arrhythmia. For a medical device to be used or “prescribed” by a healthcare professional, it should demonstrate safety and reliability, validity and (ideally) come with evidence of effectiveness. Value for money from the healthcare system perspective is also likely to be necessary if the cost of the device (and the associated monitoring) is to be reimbursed rather than bought by the patient.

Safety and effectiveness are typically evaluated by regulatory bodies such as the Food and Drug Administration (FDA) in the USA or by national bodies such as the Medicines and Healthcare Products Regulatory Agency (MHRA) in the UK. Clinical and cost-effectiveness is judged by a variety of bodies, depending on the healthcare system and key payors (insurance, occupational, state), but may include national health technology assessment (HTA) bodies such as the National Institute for Health and Care Excellence (NICE) In England. The evidence frameworks for evaluation of digital technologies are in rapid evolution, e.g. NICE has recently published an evidence standards framework for digital technologies, outlining the goals new technologies must meet in order to meet criteria for approval [7], and other countries are adopting similar, but not identical, approaches. It would appear that the trend for new technologies to be launched in a market without clinical evidence of “value” in the broadest sense is reducing, with increasing realisation that ultimately evidence is required for healthcare decisions to be made.

The value equation for devices marketed directly at consumers is more complex and also evolving. In general, if the device might provide data that would influence medical decision-making, it requires to conform to higher standards, but if it only makes claims to support health and lifestyle decision-making, it may be able to access the consumer market provided it is safe, functions as it claims to function and does not make claims that cannot be supported. From the healthcare and healthcare professional perspective, data from such devices is increasingly being “offered” to clinicians by patients, and it can be difficult for that clinician to assess the likely validity, or to incorporate the data into the traditional electronic medical record, and to robustly document how the data has or has not influenced decision-making. Concern regarding validity and legal liability may mean that the healthcare professional ignores the data, leading to the patient feeling disempowered or rather distanced from the decision-making processes [8••]. Rarely does a healthcare professional know which consumer device to recommend, except for more “medical” devices such as Kardia™ for smartphone arrhythmia detection or home blood pressure monitoring devices. The additional time and opportunity cost (or fear of this) of dealing with remotely collected data from a “consumer” device may also act as a barrier to adoption. Where there is remuneration for the clinician or the health system, adoption is likely to be much more rapid, as has been seen with a variety of home telemonitoring systems for HF in France since 2014 when the “ETAPES” government initiative was rolled out and subsequently renewed in 2018 [9].

## Direct-to-Consumer Devices and their Potential Use

Most wearable technology is marketed directly to consumers who are typically healthy (and of younger age than the average HF patient—which is around 80 years of age in high-income countries such as the UK). The trend to improved precision, accuracy and reliability of such devices may mean that, despite the restrictions placed on what the manufacturers can claim, the data may be used in decision-making by healthcare professionals.

### Activity Monitors

The most common consumer wearables are activity monitors, in the form of a “smart” watch, activity band or pedometer. The device houses an accelerometer that measures movement, often at the level of the wrist. An algorithm converts the movement data into estimates of physical activity. There are many commercially available devices, with different hardware and algorithms.

Kooiman and colleagues compared 10 activity monitors in healthy volunteers. In laboratory conditions (i.e. walking on a treadmill) most showed very high accuracy in estimating step count, with 5 out of 10 devices tested having < 1% error deviation [10]. The same devices tested in “free-living” conditions were found to have generally good agreement, albeit with larger confidence intervals than in laboratory conditions. An important limitation of accelerometry is that the type of movement influences the accuracy of the monitor; devices were found to be less accurate at slow ambulation speeds [11] which is particularly relevant for patients with HF. In a study in older patients, wrist-worn activity monitors consistently undercounted steps at low speeds, and overcounted during sedentary activity, particularly if monitors were worn on the dominant hand [12].

### Heart Rate Monitors

Most wrist-worn activity monitors are also able to monitor heart rate via photoplethysmography (PPG). This uses a light-emitting diode (LED) to illuminate a capillary bed and thus monitor for pulsatile changes in light absorption, in the same way as a pulse oximeter [13]. The accuracy of wearable PPG heart rate monitors is dependent on the type of activity being performed and the heart rate. Performance is variable on exertion, even in healthy people; a study of four wearable PPG wristbands compared with an ECG during treadmill exercise showed 95% limits of agreement of ± 20 bpm for every monitor tested [14]. This was despite the fact that in a separate study, the exercise with the most accurate heart rate measurement was running on a treadmill, whilst cycling showed the greatest error [15].

A study specifically in heart failure patients using a Fitbit™ and Apple™ watch showed poor accuracy in measuring dynamic heart rate changes [16], and so heart rate monitor data is likely best used for measuring resting heart rates.

### Facilitating Exercise and Rehabilitation

Activity monitors have typically been used by healthy adults to track and support fitness goals, but the technological ability to accurately measure exertion means that such wearables could be useful tools for supporting activity and rehabilitation in people living with HF. Exercise training reduces the risk of hospitalisation and improves exercise capacity and quality of life [17] and is a class I recommendation (“should do”) of the European Society of Cardiology and American College of Cardiology [18, 19]. A lack of facilities and/or trained staff, and low patient adherence, often limits the wider uptake of exercise training in HF [20]. Wearable activity monitors have therefore been suggested as a potential solution to such problems [21].

A sub-study of the Teledi@log Trial demonstrated a significant increase in activity using telephone-based cardiac rehabilitation with Fitbit™ activity monitors; mean step count increased from a baseline of 5899 steps per day to 7890 steps per day after 1 year [22]. Twenty per cent of these patients had HF. Even in this research study, there was a significant patient dropout, and those with lower step counts were less likely to continue using the device.

### Monitoring Response to Treatment

Activity monitors can be used as a research tool to measure response to treatment in a quantitative manner, without the use of questionnaires. A double-blind crossover trial of isosorbide mononitrate (ISMN) in patients with HF with preserved ejection fraction (HFPEF) reported that nitrate prescription was associated with lower activity in a dose-related response [23]. A study is underway to assess the effect of sacubitril/valsartan prescription on activity levels compared with enalapril [24]. Similarly, heart rate monitors could be used to titrate negatively chronotropic heart failure medications such as β-blockers, with the advantage of having continuous data rather than a single clinic visit “snapshot” and perhaps obviating the need for at least some of the face-to-face clinic reviews, thus increasing patient (and family) convenience, reducing healthcare costs, improving clinical efficiency and reducing the carbon footprint of healthcare facility visits.

### Symptom Monitoring and Prognostication

The functional classification of heart failure (such as by New York heart Association [NYHA] Class) is subjective, and there may be a marked difference between the assessment by a healthcare professional and the patient; assessment is often based on brief clinical encounters; and self-reported walking distance corresponds poorly with exercise testing [25]. Six-minute walk tests are rarely carried out except in clinical trials, and formal cardiopulmonary exercise testing is performed in only a very small minority of patients as it is expensive and time-consuming. Activity monitors could therefore be used to provide a more objective measure of functional limitation and one that relates more closely to the individual’s usual activity patterns. A study of 50 heart failure patients with activity monitors measured “free-living” step counts over 2 weeks [26]. Mean step counts were significantly different between NYHA classes, although there was some overlap in activity levels between the more sedentary class II and more active class III participants.

Cohort studies have sought to examine the prognostic value of measuring physical activity in HF via accelerometers. A retrospective study of 189 American patients with a self-reported HF diagnosis found that physical activity measured by accelerometry was strongly associated with mortality; for every 60 min of additional physical activity each day, patients with HF had a 35% reduced risk of mortality [27]. A prospective study of 170 Japanese heart failure patients showed that step counting via a waist-worn activity monitor, VO_2_max and VE/VCO_2_ slope on exercise testing were independently associated with mortality [28]. A step count < 4889 steps/day was the strongest independent predictor of mortality with a hazard ratio of 2.28 on multivariable analysis over a median follow-up of 3.7 years. There is currently no gold standard for activity assessment or grading: different studies have used different measurements of physical activity (e.g. step count, device-generated arbitrary units, duration of activity) [29]. The impact of such assessments on decision-making and quality of life, or indeed on disease or illness trajectory, is unknown. Currently we are not aware of any clinical HF service that routinely uses wearables to assess patient activity.

### Improving Blood Pressure Control

Hypertension is a common cause or contributor to heart failure, and hypotension is a common result of heart failure and its treatment. Therapy adjustments are often triggered by blood pressure readings, typically from one-off assessments at a clinic or home visit. At least one miniature smartwatch-integrated sphygmomanometer (Omron™ HeartGuide) has met the American National Standards Institute criteria for measuring blood pressure by oscillometry across a range of blood pressures; compared with manual measurement, the mean differences were − 0.9 ± 6.8 mmHg for systolic blood pressure and − 1.1 ± 5.5 mmHg for diastolic pressure [30]. The use of such a wearable device may facilitate optimal adjustment of antihypertensive or heart failure medication, monitoring of iatrogenic hypotension, and support persistence with therapy, but such impacts remain to be demonstrated.

### Arrhythmia Detection

Atrial fibrillation (AF) is common in HF [31], and the detection of such an arrhythmia would typically trigger a strong recommendation for oral anticoagulation and consideration of changes to other drug therapies [18]. Beat-to-beat variation in PPG waveforms can be analysed by algorithms to detect an irregular pulse [32]. Ascertaining the exact cause of an irregular pulse, however, is challenging with PPG alone [33] and typically requires electrocardiographic confirmation. Ambulatory ECG monitoring traditionally required a Holter monitor but can now be performed via a wearable patch. Two such devices were recently approved by the FDA: the Carnation™ patch and VivaLNK™, both of which can be worn for several days at a time for continuous ECG monitoring more comfortably and for longer than a standard Holter monitor. Electrodes can also be woven into a “smart textile” vest for prolonged multi-lead ECG monitoring [34], e.g. Cardioskin™. ECG monitoring through wearables can therefore complement PPG for the diagnosis of AF.

The Apple™ Heart Study investigated whether its smartwatch PPG-based algorithm for detecting irregular pulses could be used to screen for AF in patients with no known history of AF [35••]. 0.5% of participants received an irregular pulse notification and were then asked to initiate a telemedicine consultation, after which they would be sent a 7-day wearable ECG patch. Although the majority of patients (56%) with an irregular pulse notification did not initiate a consultation, 34% of those who did and then received and returned their patch had an episode of AF confirmed during the 7-day ECG recording. Perhaps more interestingly, 71% of irregular pulse notifications occurring during simultaneous ECG patch recording were confirmed to be AF; the

false positives mostly comprised premature atrial contractions. Much like AF detected through implantable devices, however, it is still uncertain how we use this data [36]; how long an episode of AF should prompt anticoagulation is still a matter of debate. To address this, a recently announced “pragmatic” randomised trial aims to enrol 150,000 patients with an Apple™ watch to investigate the effect of detecting AF through the device on all-cause mortality, stroke, anticoagulation usage and health resource utilisation amongst other things (NCT04276441).

PPG is less well studied for diagnosing other arrhythmia. Sudden sustained increases in heart rate can indicate paroxysmal tachycardia [37]; feasibly a similar approach of ECG patch testing following a sudden tachycardia alert could be studied for the detection of ventricular tachycardia in heart failure patients, which could provide more data for decisions regarding defibrillator therapy.

Certain smartwatches (such as the Apple™ watch and the Verily™ Study watch) have built-in electrodes allowing the generation of an ECG. In the case of the Apple™ watch, the electrodes are on the base of the watch (in contact with the wrist) and on the crown; touching the crown with a finger of the opposite hand produces a lead I ECG. By moving the watch to different locations, it is possible to generate other standard vectors, creating a 6-lead [38] or even 12-lead ECG [39], though of course the patient would not be expected to know how to do this themselves. This could, however, be used for remote consultations with clear instruction. The Apple™ watch’s ECG functionality has been cleared by the FDA for the detection of AF, but it has not yet been studied in other arrhythmia; it could potentially be used to capture patient-activated rhythm strips to assist with the diagnosis of palpitations, although such usage is not suggested by the manufacturer.

### Earlier Detection of Decompensation

The need for urgent HF hospitalisation is a serious event; in-hospital mortality rates range from 4 to 10% [40]; and mean costs of admission, which vary with healthcare system, are estimated at £2274 in the UK [41] and $14,631 in the USA [42]. There has, therefore, been considerable focus on developing technologies to support earlier detection of decompensation, with the hope that measures can be initiated to re-stabilise the syndrome and avoid the need for hospitalisation. Pulmonary artery pressure monitoring [5•] and implantable pacemaker device algorithms [43•] have some value in identifying high risk of decompensation, and wearable devices are now being developed as a non-invasive alternative to such technologies. Intrathoracic impedance can be a biomarker for pulmonary congestion and impending decompensation [44] and has been used as part of predictive algorithms in implantable devices [43•, 45]. Wearable vests that measure intrathoracic impedance have shown good correlation with fluid status [46, 47]. An algorithm using non-invasive intrathoracic impedance had a sensitivity of 60% and specificity of 96% for predicting heart failure decompensation in an observational study of 91 patients [48]. A similar study using a different vest on 106 participants applied an automated algorithm tailored to each patient, with an overall sensitivity of 87% and a specificity of 70% for decompensation [49]. There is always a trade-off between the false-positive and false-negative rates, and we await data to see whether wearables can be useful in admissions avoidance, given that device-based impedance monitoring without other parameters actually increased heart failure hospitalisation in one randomised trial, likely due to patient, family and physician concern caused by an audible “alert” being triggered in the implantable device [50]. To overcome the limitations of single channel monitoring, multiparameter monitoring wearable devices are being investigated. The LINK-HF study used a multiparameter patch sensor with ECG monitoring, intrathoracic impedance detection, accelerometry and temperature sensor on 100 patients with heart failure [51••]. A machine learning algorithm was then used on the data to create a personalised baseline for each individual, and then, a further predictive algorithm for decompensation was derived which demonstrated a sensitivity of 76% and specificity of 85% for a decompensation alert 10 days before the event. This is higher than the 70% sensitivity reported by the MultiSENSE trial of invasive device-based multiparameter monitoring [43•], which is now being tested in a large two-phase randomised trial (NCT03237858). A technology closely related to intrathoracic impedance monitoring is dielectric sensing, which is applying a low-power electromagnetic impulse across tissue to estimate its water content [52]. Remote dielectric sensing (ReDS™) is a wearable vest being investigated for use in heart failure. An observational study of 50 patients showed an 87% reduction in hospitalisation with ReDS™-directed medical titration compared with the 90 days prior to enrolment and a subsequent 79% increase in hospitalisations in the 90 days following removal of the vest [53••]. These promising early results are being followed up with an ongoing clinical trial (NCT03586336).

### Technology in Development for Early Detection of HF Decompensation

The ZOLL μCor™ is an FDA-approved patch with an ECG monitor and radiofrequency sensor and transmitter designed to measure pulmonary fluid content and is currently being evaluated in a clinical trial for predicting heart failure decompensation (NCT03476187). Seismocardiography is the measurement of chest wall vibrations correlating with the movement of the heart in the chest and blood flow through the arterial tree [54]. In an observational study of 45 heart failure patients wearing a seismocardiography patch, graphic analysis was able to differentiate between compensated and decompensated patients’ responses to a 6-min walk test and importantly within-subject improvements correlated with changes in seismocardiography measurements [55]. Furthermore, incorporating measurements from multiple seismocardiogram sensors integrated into a wearable vest, algorithms were able to predict real-time left ventricular ejection fraction [56]. Multiple electrodes imbedded in smart textiles could allow extraction of information such as heart rate, heart rate variability, respiratory rate and thoracic impedance, comparable with device-based algorithms such as HeartLogic™ [43•]. A study is underway to see to what extent these textile-based sensors can predict heart failure decompensation (NCT03719079). Finally, a prototype “smart” sock has been developed for detection of oedema and activity, with built-in accelerometer and stretch sensor. This showed high accuracy for measuring ankle circumference and physical activity in healthy subjects [57].

## Data Governance and Device Regulation

Wearable devices work by generating data; a key issue is who owns and can access these data and what consent process is visible to the patient (and healthcare professional). The data generated by wearables are valuable for technology companies as they provide insights into consumer behaviour and product use and be mined to refine algorithms and target marketing. As the data collected become more granular, it becomes more likely to be identifiable: for example, an individual’s movement data and physical location are quite likely to be unique, even if anonymised. The resulting dataset could foreseeably lead to an identifiable “digital fingerprint” for every user. In the USA, medical information is regulated by the Health Insurance Portability and Accountability Act (HIPAA). However, this focuses on insurance companies and healthcare providers, and legislation has yet to catch up with the proliferation of the wearable market; a technology manufacturer is not included as a “covered entity”; and regulations would likely only apply when the data interface with health records. In the EU, the General Data Processing Regulation (GDPR) is much stricter and mandates a Data Protection Impact Assessment for all technologies that process personal data. For this reason, many technology companies store user data outside of the EU. A related issue is whether wearables are consumer products or medical devices. The EU Regulation on Medical Devices, which has recently superseded the old Medical Devices Directive, sets a high bar for providing a “CE mark” indicating its approval. Many manufacturers are therefore careful to avoid making medical claims and instead market their products for fitness, health and “wellness”. This passes legal liability on to the physician if they choose to use it to inform medical decision-making or the use of other medical technologies. A summary of some of the other challenges to technology adoption is given in Table 1.

**Table 1:** Barriers and solutions to the large-scale deployment of digital health-based care in cardiology

Barriers to deployment	Issues	Solutions
Stakeholder resistance to adopt digital care	• Lack of patient motivation and digital skills • Lack of healthcare provider belief in digital healthcare	• Patient digital health education programmes • Redesign contemporary workflow models
Legal, ethical and technical barriers	• Privacy, security and liability concerns • Lack of interoperability	• European-wide digital health certification programmes • Assure compliance to digital health directives • Assure interoperability of digital health services
Lack of reimbursement	• Lack of health economical evaluation	• Encourage economical evaluations of digital health-based care • Inform health insurance industry and policy makers • Stimulate digital health-related knowledge and experience sharing

Adapted from Frederix et al.

## Conclusions

The field of wearable devices is rapidly evolving. The majority of currently available wearables is marketed directly to consumers for fitness tracking, but the improving precision of the instruments means they may be useful tools in decision-making about HF care. Better lifestyle choices and disease prevention are likely to be supported by development in wearable technology and information interfaces with consumers, patients and healthcare professionals. The future will bring better identification of wearable technology that can inform medical decision-making and support better self- and remote monitoring and more personalised surveillance and adjustment of therapy to improve the outcome and experience of care. Many barriers still exist to the easy integration of wearable technology and the derived data into the healthcare system—but key stakeholders such as governments, regulators and reimbursement authorities are keen to facilitate the appropriate identification and implementation of digital solutions to modern healthcare needs.

## References

[1] Mosterd A, Hoes AW (2007). Clinical epidemiology of heart failure. Heart..

[2] Bleumink GS, Knetsch AM, Sturkenboom MCJM, Straus SMJM, Hofman A, Deckers JW (2004). Quantifying the heart failure epidemic: prevalence, incidence rate, lifetime risk and prognosis of heart failure - the Rotterdam Study. Eur Heart J.

[3] Conrad N, Judge A, Tran J, Mohseni H, Hedgecott D, Crespillo AP, Allison M, Hemingway H, Cleland JG, McMurray JJV, Rahimi K (2018). Temporal trends and patterns in heart failure incidence: a population-based study of 4 million individuals. Lancet..

[4] Maggioni AP, Dahlström U, Filippatos G, Chioncel O, Leiro MC, Drozdz J, Fruhwald F, Gullestad L, Logeart D, Fabbri G, Urso R, Metra M, Parissis J, Persson H, Ponikowski P, Rauchhaus M, Voors AA, Nielsen OW, Zannad F, Tavazzi L, on behalf of the Heart Failure Association of the European Society of Cardiology (HFA) (2013). EURObservational research programme: regional differences and 1-year follow-up results of the heart failure pilot survey (ESC-HF pilot). Eur J Heart Fail.

[5] Abraham WT, Stevenson LW, Bourge RC, Lindenfeld JA, Bauman JG, Adamson PB (2016). Sustained efficacy of pulmonary artery pressure to guide adjustment of chronic heart failure therapy: complete follow-up results from the CHAMPION randomised trial. Lancet.

[6] Gallup News Service. Gallup Poll Social Series: Health and Healthcare. Available from: https://news.gallup.com/poll/269096/one-five-adults-health-apps-wearable-trackers.aspx. Published November 2019. Accessed February 18, 2020.

[7] NICE. National Institute for Health and Care Excellence Evidence Standards Framework for Digital. 2019;(March):35.

[8] Frederix I, Caiani EG, Dendale P, Anker S, Bax J, Böhm A (2019). ESC e-cardiology working group position paper: overcoming challenges in digital health implementation in cardiovascular medicine. Eur J Prev Cardiol.

[9] ÉTAPES: Expérimentations de Télémédecine pour l’Amélioration des Parcours En Santé. Available from: https://solidarites-sante.gouv.fr/soins-et-maladies/prises-en-charge-specialisees/telemedecine/article/etapes-experimentations-de-telemedecine-pour-l-amelioration-des-parcours-en. Accessed March 4, 2020.

[10] Kooiman TJM, Dontje ML, Sprenger SR, Krijnen WP, van der Schans CP, de Groot M (2015). Reliability and validity of ten consumer activity trackers. BMC Sports Sci Med Rehabil.

[11] Feehan LM, Geldman J, Sayre EC, Park C, Ezzat AM, Young Yoo J, et al. Accuracy of fitbit devices: systematic review and narrative syntheses of quantitative data. JMIR Mhealth Uhealth. 2018;6(8):e10527. 10.2196/10527 PMC6107736 30093371

[12] Tedesco S, Sica M, Ancillao A, Timmons S, Barton J, O’Flynn B (2019). Accuracy of consumer-level and research-grade activity trackers in ambulatory settings in older adults. PLoS One.

[13] Allen J. Photoplethysmography and its application in clinical physiological measurement. Physiol Meas. 2007;28(3):R1–39. 10.1088/0967-3334/28/3/R01 17322588

[14] Cadmus-Bertram L, Gangnon R, Wirkus EJ, Thraen-Borowski KM, Gorzelitz-Liebhauser J (2017). The accuracy of heart rate monitoring by some wrist-worn activity trackers. Ann Intern Med.

[15] Kondama Reddy R, Pooni R, Zaharieva DP, Senf B, El Youssef J, Dassau E, et al. Accuracy of wrist-worn activity monitors during common daily physical activities and types of structured exercise: evaluation study. JMIR mHealth uHealth. 2018;6(12):e10338. 10.2196/10338 PMC6305876 30530451

[16] Moayedi Y, Abdulmajeed R, Duero Posada J, Foroutan F, Alba AC, Cafazzo J, Ross HJ (2017). Assessing the use of wrist-worn devices in patients with heart failure: feasibility Study. JMIR Cardio.

[17] Taylor RS, Sagar VA, Davies EJ, Briscoe S, Coats AJS, Dalal H (2014). Exercise-based rehabilitation for heart failure. Cochrane Database Syst Rev.

[18] Ponikowski P, Voors AA, Anker SD, Bueno H, Cleland JGF, Coats AJS (2016). ESC guidelines for the diagnosis and treatment of acute and chronic heart failure. Eur Heart J.

[19] Yancy CW, Jessup M, Bozkurt B, Butler J, Casey DE, Drazner MH (2013). 2013 ACCF/AHA guideline for the management of heart failure: a report of the American College of Cardiology Foundation/American Heart Association task force on practice guidelines. J Am Coll Cardiol.

[20] Piepoli MF, Conraads V, CorrÁ U, Dickstein K, Francis DP, Jaarsma T (2011). Exercise training in heart failure: from theory to practice. A consensus document of the Heart Failure Association and the European Association for Cardiovascular Prevention and Rehabilitation. Eur J Heart Fail.

[21] Alharbi M, Straiton N, Gallagher R (2017). Harnessing the potential of wearable activity trackers for heart failure self-care. Curr Heart Fail Rep.

[22] Thorup C, Hansen J, Grønkjær M, Andreasen JJ, Nielsen G, Sørensen EE, Dinesen BI (2016). Cardiac patients’ walking activity determined by a step counter in cardiac telerehabilitation: data from the intervention arm of a randomized controlled trial. J Med Internet Res.

[23] Redfield MM, Anstrom KJ, Levine JA, Koepp GA, Borlaug BA, Chen HH, LeWinter MM, Joseph SM, Shah SJ, Semigran MJ, Felker GM, Cole RT, Reeves GR, Tedford RJ, Tang WHW, McNulty SE, Velazquez EJ, Shah MR, Braunwald E (2015). Isosorbide mononitrate in heart failure with preserved ejection fraction. N Engl J Med.

[24] Khandwalla RM, Birkeland K, Heywood JT, Steinhubl S, McCague K, Fombu E, Grant D, Riebman JB, Owens RL (2019). Activity sensors to evaluate the effect of sacubitril/valsartan on quality-of-life in heart failure: rationale and design of the AWAKE-HF study. ESC Heart Fail.

[25] Raphael C, Briscoe C, Davies J, Whinnett ZI, Manisty C, Sutton R (2007). Limitations of the New York Heart Association functional classification system and self-reported walking distances in chronic heart failure. Heart..

[26] Baril JF, Bromberg S, Moayedi Y, Taati B, Manlhiot C, Ross HJ, et al. Use of free-living step count monitoring for heart failure functional classification: Validation study. J Med Internet Res. 2019;3(1):e12122. 10.2196/12122 PMC6834224 31758777

[27] Loprinzi PD (2016). The effects of free-living physical activity on mortality after congestive heart failure diagnosis. Int J Cardiol.

[28] Izawa KP, Watanabe S, Oka K, Hiraki K, Morio Y, Kasahara Y, Brubaker PH, Osada N, Omiya K, Shimizu H (2013). Usefulness of step counts to predict mortality in Japanese patients with heart failure. Am J Cardiol.

[29] Tan MKH, Wong JKL, Bakrania K, Abdullahi Y, Harling L, Casula R, Rowlands AV, Athanasiou T, Jarral OA (2019). Can activity monitors predict outcomes in patients with heart failure? A systematic review. Eur Heart J Qual Care Clin Outcomes.

[30] Kuwabara M, Harada K, Hishiki Y, Kario K (2019). Validation of two watch-type wearable blood pressure monitors according to the ANSI/AAMI/ISO81060-2:2013 guidelines: Omron HEM-6410T-ZM and HEM-6410T-ZL. J Clin Hypertens.

[31] Anter E, Jessup M, Callans DJ (2009). Atrial fibrillation and heart failure: treatment considerations for a dual epidemic. Circulation..

[32] Paradkar N, Chowdhury SR. Cardiac arrhythmia detection using photoplethysmography. Conf Proc IEEE Eng Med Biol Soc. 2017;2017:113–16. 10.1109/EMBC.2017.8036775 29059823

[33] Chan PH, Wong CK, Poh YC, Pun L, Leung WWC, Wong YF (2016). Diagnostic performance of a smartphone-based Photoplethysmographic application for atrial fibrillation screening in a primary care setting. J Am Heart Assoc.

[34] Vardas P, Cowie M, Dagres N, Asvestas D, Tzeis S, Vardas EP, Hindricks G, Camm J (2020). The electrocardiogram endeavour: from the Holter single-lead recordings to multilead wearable devices supported by computational machine learning algorithms. Europace..

[35] Perez MV, Mahaffey KW, Hedlin H, Rumsfeld JS, Garcia A, Ferris T (2019). Large-scale assessment of a smartwatch to identify atrial fibrillation. N Engl J Med.

[36] Mulder BA, Van Gelder IC, Rienstra M (2019). Device-detected atrial fibrillation. Circulation..

[37] Ip JE (2019). Wearable devices for cardiac rhythm diagnosis and management. JAMA..

[38] Samol A, Bischof K, Luani B, Pascut D, Wiemer M, Kaese S. Single-lead ECG recordings including einthoven and wilson leads by a smartwatch: a new era of patient directed early ECG differential diagnosis of cardiac diseases? Sensors (Switzerland). 2019;19(20):4377. 10.3390/s19204377 PMC6832209 31658713

[39] Cobos Gil MÁ. Standard and precordial leads obtained with an Apple watch. Ann Intern Med. 2020;172(6):436–37. 10.7326/M19-2018 31766051

[40] Cowie MR, Anker SD, Cleland JGF, Felker GM, Filippatos G, Jaarsma T, Jourdain P, Knight E, Massie B, Ponikowski P, López-Sendón J (2014). Improving care for patients with acute heart failure: before, during and after hospitalization. ESC Heart Fail.

[41] NICE (National Institute for Health and Care Excellence). Putting NICE guidance into practice: Chronic heart failure in adults: diagnosis and management (NG106). Published September 2018. https://www.nice.org.uk/guidance/ng106/resources/resourceimpact-report-pdf-6537494413. Accessed February 26, 2020. 30645061

[42] Kilgore M, Patel HK, Kielhorn A, Maya JF, Sharma P (2017). Economic burden of hospitalizations of Medicare beneficiaries with heart failure. Risk Manag Healthc Policy.

[43] Boehmer JP, Hariharan R, Devecchi FG, Smith AL, Molon G, Capucci A (2017). A multisensor algorithm predicts heart failure events in patients with implanted devices: results from the MultiSENSE study. JACC Heart Fail.

[44] Abraham WT (2007). Intrathoracic impedance monitoring for early detection of impending heart failure decompensation. Congest Heart Fail.

[45] Ypenburg C, Bax JJ, van der Wall EE, Schalij MJ, van Erven L (2007). Intrathoracic impedance monitoring to predict decompensated heart failure. Am J Cardiol.

[46] Gastelurrutia P, Cuba-Gyllensten I, Lupon J, Zamora E, Llibre C, Caballero Á, Riistama J, Aarts R, Bayes-Genis A (2016). Wearable vest for pulmonary congestion tracking and prognosis in heart failure: a pilot study. Int J Cardiol.

[47] Lee S, Squillace G, Smeets C, Vandecasteele M, Grieten L, De Francisco R (2015). Congestive heart failure patient monitoring using wearable bio-impedance sensor technology. Conf Proc IEEE Eng Med Biol Soc.

[48] Cuba Gyllensten I, Bonomi AG, Goode KM, Reiter H, Habetha J, Amft O, Cleland JGF (2016). Early indication of decompensated heart failure in patients on home-telemonitoring: a comparison of prediction algorithms based on daily weight and noninvasive transthoracic bio-impedance. JMIR Med Inform.

[49] Darling CE, Dovancescu S, Saczynski JS, Riistama J, Sert Kuniyoshi F, Rock J, Meyer TE, McManus DD (2017). Bioimpedance-based heart failure deterioration prediction using a prototype fluid accumulation vest-mobile phone dyad: an observational study. JMIR Cardio.

[50] Van Veldhuisen DJ, Braunschweig F, Conraads V, Ford I, Cowie MR, Jondeau G (2011). Intrathoracic impedance monitoring, audible patient alerts, and outcome in patients with heart failure. Circulation..

[51] Stehlik J, Schmalfuss C, Bozkurt B, Nativi-Nicolau J, Wohlfahrt P, Wegerich S (2020). Continuous wearable monitoring analytics predict heart failure hospitalization: the LINK-HF multicenter study. Circ Heart Fail.

[52] Amir O, Rappaport D, Zafrir B, Abraham WT (2013). A novel approach to monitoring pulmonary congestion in heart failure: initial animal and clinical experiences using remote dielectric sensing technology. Congest Hear Fail.

[53] Amir O, Ben-Gal T, Weinstein JM, Schliamser J, Burkhoff D, Abbo A (2017). Evaluation of remote dielectric sensing (ReDS) technology-guided therapy for decreasing heart failure re-hospitalizations. Int J Cardiol.

[54] Inan OT, Migeotte P, Park K, Etemadi M, Tavakolian K, Casanella R (2015). Ballistocardiography and seismocardiography: a review of recent advances. IEEE J Biomed Health Inform.

[55] Inan OT, Baran Pouyan M, Javaid AQ, Dowling S, Etemadi M, Dorier A (2018). Novel wearable seismocardiography and machine learning algorithms can assess clinical status of heart failure patients. Circ Heart Fail.

[56] Lin WY, Ke HL, Chou WC, Chang PC, Tsai TH, Lee MY. Realization and technology acceptance test of a wearable cardiac health monitoring and early warning system with multi-channel MCGs and ECG. Sensors (Switzerland). 2018;18(10):3538. 10.3390/s18103538 PMC6210769 30347695

[57] Fallahzadeh R, Pedram M, Ghasemzadeh H (2016). SmartSock: a wearable platform for context-aware assessment of ankle edema. Conf Proc IEEE Eng Med Biol Soc.

